# A Review of Tubal Factors Affecting Fertility and its Management

**DOI:** 10.7759/cureus.30990

**Published:** 2022-11-01

**Authors:** Ketki Ambildhuke, Sandhya Pajai, Anjali Chimegave, Radhika Mundhada, Priyanka Kabra

**Affiliations:** 1 Obstetrics and Gynecology, Jawaharlal Nehru Medical College, Datta Meghe Institute of Medical Sciences, Wardha, IND; 2 Community Medicine, Jawaharlal Nehru Medical College, Datta Meghe Institute of Medical Sciences, Wardha, IND; 3 Medicine, Jawaharlal Nehru Medical College, Datta Meghe Institute of Medical Sciences, Wardha, IND

**Keywords:** tubal patency, obstetric usg, hysterosalpingography, tubal cannulation, infertility, fallopian tube

## Abstract

Infertility is a problem that affects both developed and developing countries today. Many couples choose to have financial stability before conception, irrespective of age. Tubal blockage accounts for 30%-40% of a woman’s fertility. Congenital abnormalities, acute and persistent inflammatory diseases, endometriosis, and different pathologies are associated with infertility and cause partial or complete obstruction of the fallopian tubes. Approximately 30% of women experience infertility due to fallopian tube illness, with 10%-25% of these women experiencing proximal fallopian tube obstruction. The fallopian tube is an integral part of the union of sperm, and its normal function is a prerequisite for natural conception. Tubal obstruction is a common cause of infertility. These patients are keen to unblock their blocked fallopian tubes and restore reproductive function. Accurate diagnosis and optimal treatment options are essential for treating infertility.

## Introduction and background

Procreation, or the desire to have one's own offspring, is the greatest desire among human beings. Infertility is more of a social and mental health problem that affects a person and their family's personal, social, and mental health [1]. It is estimated that 10%-15% of married couples suffer from infertility. Due to changing lifestyles, social systems, professional life, and academic achievements, more and more couples face this problem. According to the World Health Organization (WHO), a nation with complete physical, intellectual, and social well-being has a woman with good reproductive health. Infertility implies an apparent failure of a pair to conceive, even if sterility shows absolute incapacity to conceive for one or greater reasons. If a couple has not conceived after one year of normal and unprotected intercourse, it means that the couple needs to be investigated. It has been brought to light that 80% of regular couples conceive within a year. It has been discovered that tubal damage results in tubal blockage in 12% of cases. The incidence will increase to 23% after a pelvic inflammatory illness. The fallopian tube is a muscular tube that connects the ovary and the uterus and is divided into the following areas: interstitial, isthmus, ampulla, and fimbrial end. The fallopian tube is an integral part of sperm attachment and fertilization, and its normal function is necessary for natural conception. Blockage of the fallopian tubes is a common disease and one of the leading causes of infertility [2]. Inflammation is the primary cause of oviduct obstruction, and infections of the reproductive system increase the source of infection. Infertile women with blocked fallopian tubes and hydrosalpinx account for 30% to 40% of the entire infertile population. [3,4]. Numerous pathological situations related to infertility have been validated to damage cilia or lessen ciliary motion. Mere patency of the tubal lumen isn't the simplest way to affect fertility. The endosalphinx is covered by ciliated epithelial cells and secretory cells. The cilia allow for the propulsion of the fertilized egg in the direction of the uterine cavity. The secretory cells offer vitamins to the sperm in addition to the ovum throughout their passage through the tube. The peristaltic actions of the fallopian tubes are under the effect of estrogen, progesterone, and prostaglandins, and synchronized movements allow for the propulsion of sperm and fertilized eggs in both directions. The ovarian fimbriae bring the ovum into the fimbrial end at the time of ovulation.

## Review

Etiology

Recent research has shown that the most common cause of tubal factor infertility is obstruction of the fallopian tubes due to sexually transmitted infection with Chlamydia trachomatis or Neisseria gonorrhoeae causing salpingitis [5]. Other factors affecting tubal patency are pelvic infections, such as adhesions around the fallopian tube due to abdominal tuberculosis; injury to the fallopian tube due to previous tubal surgeries or sterilization; ischemic nodules; endometriosis; polyps or mucus; tubal spasm; congenitally abnormal tubes. Peritoneal factors such as peritubular adhesions, endometriosis, altered tubal motility, and fimbrial end blockage also affect tubal patency.

Investigations

Hysterosalpingography (HSG) can be performed as a preliminary screening test in the case of suspected fallopian tube infertility in women with no pelvic inflammatory disease or endometriosis. But if a hysterosalpingo-contrast-ultrasonography (HyCoSy) is available, it should be utilized instead. If there are any related comorbidities, the patient should undergo laparoscopic chromopertubation [6].

**Table 1:** Tests for tubal patency

Tests for tubal patency
Hysterosalpingography (HSG)
Dilation and insufflation test (DI)
Saline infusion sonography
Hysterosalpingo-contrast-sonography (HyCoSy)
Laparoscopy and chromopertubation
Sonohysterosalpingography
Falloscopy

Insufflation Test (Rubin’s test)

The test should be done in the postmenstrual phase from day seven to day 10 of this cycle. It should not be done in the case of a pelvic infection. Principle: a tube connects the cervical canal to the peritoneal cavity. Therefore, when pushed through the cervix and enters the peritoneal cavity under pressure, the entry of air or carbon dioxide (CO2) indicates that the fallopian tube is unobstructed (uncommon now). Observation: the patency of the tube is determined by: (a) when it rises above 120 mmHg, the pressure drops; (b) a hissing sound is heard during auscultation in any iliac fossa; (c) patient with shoulder pain (air irritates the diaphragm).

Hysterosalpingography (HSG):

It is the most commonly used method as it is non-invasive and cheap. An image intensifier should be used in the X-ray room after the radiopaque dye has been injected into the uterine cavity to inspect the fallopian tubes. A ruby cannula and a Foley catheter should be utilized to see into the uterine cavity and tubes. A late menstrual period, which is day seven to day 10 of this cycle, should be used for the test. In the case of pelvic infection, this test should not be performed. Injecting a radiopaque contrast material allows for fluoroscopic viewing of the uterine cavity and fallopian tubes during hysterosalpingography. The sensitivity is 84% and the specificity is 75% [7]. The low imaging accuracy of this method is attributed to tubal spasms, but this has been reduced by the use of intravenous scopolamine and patient switching [8]. EPI and pregnancy are two conditions that make HSG contraindicated; also, methylprednisolone 32 mg must be taken before surgery if there is a history of allergies in the patient's medical history 12 and two hours before the event. Additionally, it has been demonstrated that HSG and oily contrast agents have specific therapeutic benefits by eliminating particles from the fallopian tubes. [9]. The only disadvantage is X-ray radiation. Principle: A tube connects the cervical canal to the peritoneal cavity. Therefore, when pushed through the cervix under pressure, the radiopaque dye entering the peritoneal cavity indicates that the fallopian tube is unobstructed. Observation: Patency is confirmed by the spillage of dye bilaterally from both the Ostia. In the case of hydrosalpinx, a large mass of dye is seen without a peritoneal spill. A bilateral angle block with dye extravasation is highly indicative of tuberculous salpingitis [10].

Laproscopic Chromopertubation

Laparoscopic investigations are the gold standard for evaluating infertility caused by fallopian tube factors (permitted method). It usually takes place during the proliferation phase. It can be used to detect tubal patency, obstruction (location and size), motility, changes in hydrosalpinx, adhesions around the tubal, and fimbriae agglutination. Unfortunately, laparoscopic stained tubes remain the gold standard for tubal infertility assessment [11]. At the same time, laparoscopy was performed, and diluted methylene blue was injected into the uterine cavity to observe the fallopian tube filling and spillage into the abdominal cavity [12]. This surgery has the drawbacks of being expensive, invasive, and requiring anesthesia [13]. Laparoscopy can evaluate the structure of the fallopian tube and the relationship between the fallopian tube and other tissues and organs, accurately separate the fallopian tube adhesion and pelvic adhesion, restore the shape and movement of the fallopian tube and diagnose diseases like pelvic endometriosis. It can also be used at the same time. It increases the incidence of secondary infertility in pregnant women [14]. When hydrosalpinx under hysteroscopy indicates tubal obstruction, the guide wire can be directly recanalized and monitored laparoscopically, and has a good therapeutic effect on the proximal fallopian tube lesions [15].

**Table 2:** Indications for laparoscopy in infertility

Indications for laparoscopy in infertility
HSG reveals suspicious results
Before planning for intrauterine insemination
Before planning for ovulation induction
Hydrosalpinx removal before in-vitro fertilization in a suspected case of endometriosis

Sonohysterosalpingography

It is a useful and safe way to access the uterine cavity and check the fallopian tubes' patency [16]. Under ultrasound, about 200 ml of saline is slowly injected through an 8-number foley catheter. The condition of the fallopian tube is confirmed by the flow of saline solution along the tube and is observed by the leakage of fluid from the end of the fimbriae. Unobstructed fallopian tubes can also be confirmed by the free spillage of fluid in the pouch of Douglas [17]. The sensitivity of hysterography and hysterosalpingography to detect hydrosalpinx is 84.6% and the specificity is 99.7% [18]. Hysterosalpingo contrast sonography has progressed from 2D to 3D and even 4D imaging due to the development of contrast agents from negative saline contrast agents to positive microbubble contrast agents. [19]. HyCoSy is advantageous because patients show better pain tolerance; it avoids the use of contrast agents containing iodine and prevents the use of ionizing radiation [14].

Falloscopy

It is used to study the complete length of the fallopian tube lumen with the help of thin and flexible fiber optic equipment [20]. It is done through the uterine cavity using a hysteroscope. It helps to directly observe the fallopian tube orifice, mucosal pattern, polyps, or fragments in the fallopian tube.

Salpingoscopy

The lumen of the fallopian tube is visualized by introducing a rigid endoscope through the bristles of the tube.

Treatment of tubal infertility

Tuboplasty

**Figure 1:**
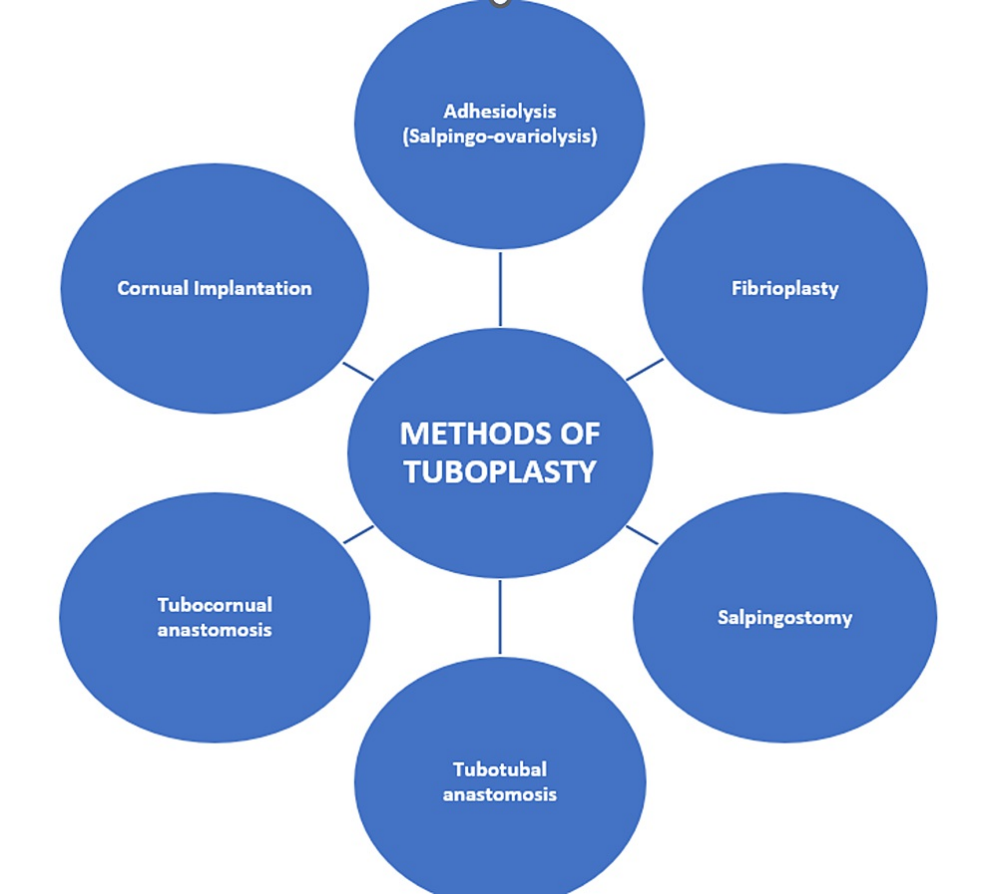
Methods of tuboplasty

Tuboplasty (tubal microsurgery) is recommended for young women with tubal blockage and prior tubal sterilization [21]. Depending upon the site of the block, several tuboplasty procedures have been performed with successful pregnancy rates varying from 27% for fimbrial surgery to 50%-60% for isthmic blockage. Salpingectomy and IVF should be done in the case of large hydrosalpinx causing distal tubal disease. Methods of tuboplasty are shown in Figure 1.

In-vitro Fertilization (IVF)

IVF is advised for women in whom tuboplasty has failed or who have extensive and irreparable tubal damage [22]. It is the best option for complicated tubal diseases. This is expensive therapy, but the prognosis is good with this. IVF is not recommended in cases of extensive pelvic adhesions and inaccessible ovaries due to adhesions; ova retrieval may be impossible or dangerous to the bowels in such cases.

Tubal cannulation under guided laparoscopy

This can be done laparoscopically under hysteroscopic guidance. In patients with hydrosalpinx when hysterosalpingography indicates tubal obstruction, tubal cannulation can be used to directly recanalize, is monitored laparoscopically, and has a good therapeutic effect on the proximal fallopian tube lesions [23]. This method has given the best results as it restores patency in 75% of cases. The test results revealed that the hysteroscopy group had a recanalization success rate of only 55%, significantly lower than the laparoscopic hysteroscopy group. However, when combined with the laparoscopic group, the recanalization success rate of patients with secondary infertility under hysteroscopy is 85%. The recanalization rate is 85.71%, which is significantly higher. For tubal cannulation, the wire is guided under laparoscopy. It is done in the lithotomy position and under general anesthesia [24]. The laparoscope is inserted into the abdominal cavity to detect the shape, size, and position of the uterus and ovaries, and to detect leakage or deformation of the fallopian tubes. At the same time, corresponding surgical operations can be performed, such as removing pelvic adhesions (if any) and restoring the normal shape of the pelvic organs as much as possible [25]. Patients with fimbriae adhesions or edema should undergo plastic surgery or ostomy; cysts of the middle fallopian tubes must be removed; endometriosis patients should undergo electrocoagulation and removal of chocolate cysts. Insert a hysteroscope to observe the thickness and any changes in the endometrium and the shape of the uterine cavity. Using a hysteroscope to insert a fallopian tube catheter through the fallopian tube hole and inject a mixed solution of methylene blue and normal saline through the catheter, one can get information about fallopian tube blockage and can be guided by laparoscopy. If the resistance is large or the fallopian tube is locally enlarged, it indicates that there is an obstruction of the fallopian tube. Recanalization of the fallopian tube involves inserting a guide wire into the fallopian tube and advancing it from the proximal end to the distal end of the fallopian tube to clear the adhesions [26]. Insert the guide wire until it reaches the fimbriae of the fallopian tube. After that, the guide wire should be taken out, and the diluted methylene blue solution should be injected again. Note that the liquid from the end of the fimbriae flows into the pouch of Douglas, indicating that the fallopian tube is patent and the blockage has been taken out. However, the solution still needs to be injected to avoid potential adhesion [27]. Under the guidance of laparoscopy, cases of unilateral or bilateral proximal fallopian tube obstruction were performed with hysteroscopic intubation of the fallopian tubes [28]. The results showed that successful incubation of the oviduct can significantly increase the rate of pregnancy. Patients with unilateral obstruction of the fallopian tube are more likely to become pregnant after successful tubal intubation [29]. This means that women with proximal fallopian tube obstruction may consider hysteroscopic intubation under laparoscopic guidance as an alternative to assisted reproductive technology [30].

Discussion

Among infertile women, 30%-40% have fallopian tube-related diseases that cause infertility. Fallopian tube disease is one of the most common factors causing female infertility and represents the need for infertility treatment. The pathogenesis is complex, including tubal inflammation, tubal tuberculosis, endometriosis, postoperative pelvic injury and adhesions, and congenital hypoplasia. One-third of tubal infertility is caused by non-specific salpingitis and is the most frequent cause of tubal blockage [31]. There are many diagnostic methods for detecting fallopian tube obstruction, such as imaging (HCG and HyCoSy), hysteroscopy, and laparoscopy. The endoscope can visually observe the state of the fallopian tube mucosa and, more importantly, is used to assess the prognosis and for therapeutic purposes. Laparoscopy is the most reliable method of inspection. However, it is expensive and invasive, but it is the most commonly used method and is a daycare surgery with minimal incision and maximum diagnostic and therapeutic value. HSG is the primary diagnostic method for obstructive tubal infertility, and sometimes it can have a therapeutic effect [32]. However, HSG means generating continuous pressure to fill the uterine cavity with a contrast agent. This situation can lead to poor patient comfort and even pain. Hysterosalpingo-contrast-sonography (HyCoSy) is also based on a principle similar to HSG, so it has similar shortcomings. Therefore, salpingography can be used for diagnosis and treatment to a large extent and can make up for the shortcomings of hysterosalpingography [33]. This article presents the incidence of infertility and its treatment with relevant clinical studies, and each has its advantages and disadvantages, indicating that hysterosalpingography can be used as a preliminary diagnosis of tubal obstruction. Laparoscopy can be used for a more detailed examination of infertility caused by diseases related to the pelvis. The choice of hysterosalpingography and interventional therapy can be used as an effective, low-cost, minimally invasive diagnosis and treatment. In addition, patients with complex conditions should be treated with a single or comprehensive treatment method depending on the specific situation to achieve good diagnostic and therapeutic results. The combined therapy approach is especially beneficial for patients suffering from secondary infertility [34]. 

## Conclusions

Fallopian tube disease is one of the most common factors causing female infertility and represents the need for infertility treatment. There are many diagnostic methods for detecting fallopian tube obstruction, such as imaging (hysterosalpingography and hysterosalpingo-contrast-sonography), hysteroscopy, and laparoscopic. This article presents the incidence of infertility and treatment of the same with relevant clinical studies, and each has its advantages and disadvantages, indicating that hysterosalpingography can be used as a preliminary diagnosis of tubal obstruction. In addition, patients with complex conditions should be treated with a single or comprehensive treatment method depending on the specific situation to achieve good diagnostic and therapeutic results. The combined technology approach is particularly effective for patients with secondary infertility.
